# Supplementary material for: Smoking Cessation Apps: A Systematic Review of Format, Outcomes, and Features
Source: Int J Environ Res Public Health. 2021 Nov 6;18(21):11664. doi: 10.3390/ijerph182111664 (PMC8583115; doi:10.3390/ijerph182111664)
Supplement: Supplementary file 1 [file ijerph-18-11664-s001.zip › Table S1.pdf]

Supplemental Material

Table S1. Literature search strategy.

	Searches PUBMED
1	(("Smoking*" OR "Tobacco Products" OR "Tobacco" OR "Tobacco Use" OR "Tobacco Use Disorder" OR “Smoke*” OR “Cigar*” OR “Nicotine” OR “Nicotine Dependence”) AND (“Cessation” OR “Prevent*” OR “Quit*” OR “Abstinence” OR “Treatment” OR “Intervention” OR “Stop” OR “Therapy” OR “Care”))
2	“Smoking Cessation”
3	“Quit Smoking”
4	“Stop Smoking”
5	“Tobacco Use Disorder”
6	"Tobacco Use Cessation Products"
7	"Tobacco Use Cessation"
8	1 OR 2 OR 3 OR 4 OR 5 OR 6 OR 7
9	“Telephone”
10	“Phone”
11	“Mobile Phone”
12	“Cell Phone”
13	“Smartphone”
14	“Smartphone app*”
15	“App” OR “Apps” OR “Application” OR “Applications”
16	“Mobile app*”
17	“Mobile health”
18	“Digital*”
19	“eHealth”
20	“e Health”
21	“e-Health”
22	“mHealth”
23	“m Health”
24	“m-Health”
25	“uHealth”
26	“u Health”
27	“u-Health”
28	“Telehealth”
29	“Tele-health”
30	“Tele health”
31	9 OR 10 OR 11 OR 12 OR 13 OR 14 OR 15 OR 16 OR 17 OR 18 OR 19 OR 20 OR 21 OR 22 OR 23 OR 24 OR 25 OR 26 OR 27 OR 28 OR 29 OR 30
32	8 AND 31
	Limit to human
	English and Spanish
	Title/Abstract

	Searches PsycINFO
1	(("Smoking*" OR "Tobacco Products" OR "Tobacco" OR "Tobacco Use" OR "Tobacco Use Disorder" OR “Smoke*” OR “Cigar*” OR “Nicotine” OR “Nicotine Dependence”) AND (“Cessation” OR “Prevent*” OR “Quit*” OR “Abstinence” OR “Treatment” OR “Intervention” OR “Stop” OR “Therapy” OR “Care”))
2	“Smoking Cessation”
3	“Quit Smoking”
4	“Stop Smoking”
5	“Tobacco Use Disorder”
6	"Tobacco Use Cessation Products"
7	"Tobacco Use Cessation"
8	1 OR 2 OR 3 OR 4 OR 5 OR 6 OR 7
9	“Telephone”
10	“Phone”
11	“Mobile Phone”
12	“Cell Phone”
13	“Smartphone”
14	“Smartphone app*”
15	“App” OR “Apps” OR “Application” OR “Applications”
16	“Mobile app*”
17	“Mobile health”
18	“Digital*”
19	“eHealth”
20	“e Health”
21	“e-Health”
22	“mHealth”
23	“m Health”
24	“m-Health”
25	“uHealth”
26	“u Health”
27	“u-Health”
28	“Telehealth”
29	“Tele-health”
30	“Tele health”
31	9 OR 10 OR 11 OR 12 OR 13 OR 14 OR 15 OR 16 OR 17 OR 18 OR 19 OR 20 OR 21 OR 22 OR 23 OR 24 OR 25 OR 26 OR 27 OR 28 OR 29 OR 30
32	8 AND 31
	Limit to human
	English and Spanish
	Abstract
	Limit to research journals (books and dissertations excluded)
