# Supplementary material for: Smoking Cessation Apps: A Systematic Review of Format, Outcomes, and Features
Source: Int J Environ Res Public Health. 2021 Nov 6;18(21):11664. doi: 10.3390/ijerph182111664 (PMC8583115; doi:10.3390/ijerph182111664)
Supplement: Supplementary file 1 [file ijerph-18-11664-s001.zip › Table S2.pdf]

Supplemental Material

Table S2. Characteristics of studies included.

Author year	Study type	Country	Definition of daily smokers	Description of smartphone app (general smartphone app or app combined face-to-face contact)	Aim	Inclusion criteria	Sample characteristics
Baskerville et al. (2018) [55]	RCT	USA	Current/daily smoker.	General smartphone app.	To determine the efficacy of CTC, on reducing smoking prevalence among young adult smokers in comparison with an evidence informed self-help guide.	(1) people aged 19–29; (2) smoked cigarettes daily; (3) resided in Canada; (4) were considering quitting smoking in the next 30 days; (5) had an Android or iPhone smartphone; (6) were able to provide informed consent; (7) were able to comprehend English; (8) and were not referred to the study by an existing study participant.	*N*=1599 (Female 45.94%); White 75.06%. CTC group (*n*=820): Female 44.9%; White 75.4%. OnRQ group (*n*=779): Female 47%; White 74.8%.
BinDhim et al. (2018) [32]	RCT	USA, Australia, Singapore and the UK	Current/daily smoker.	General smartphone app.	To assess the efficacy of SSC App with interactive smoking cessation decision-aid compared with an app that contains	(1) daily smokers of cigarettes; (2) ≥18 years old; (3) from USA, Australia, Singapore, and the UK.	*N*=684 (Female 55%; mean age 28.3; *SD*=10.0). Control group (*n*=342): Female 52.9%; mean age 28.8 (*SD*=9.8). Intervention group (*n*=342): Female 57%; mean age 27.9 (*SD*=10.2).

					only static smoking cessation information on continuous abstinence.		
Bricker et al. (2014) [33]	RCT	USA	Smoked ≥ 5 cigarettes/day for at least the past year.	General smartphone app.	To construct a randomized controlled pilot trial comparing a SmartQuit app Group (experimental group) versus QuitGuide app Group (control group) to determine: trial design feasibility; participant receptivity; satisfaction; cessation outcomes overall and for two subgroups; and the impact on acceptance of cravings.	(1) ≥18 years old; (2) smokes at least 5 cigarettes per day for at least 1 year; (3) wants to quit in the next 30 days; (4) interested in learning skills to quit smoking; (5) willing to be randomly assigned to either smartphone app; (6) US resident; (7) knows how to download a smartphone app from Apple's App Store; (8) English-reading; (9) not using other smoking cessation interventions; (10) daily access to their own Apple iPhone 4, 4s, or 5.	*N*=196. SmartQuit Group (*n*=98): Female 53%; mean age 41.5 (*SD*=12.0); Caucasian 85%; Hispanic 4%. QuitGuide Group (*n*=98): Female 51%; mean age 41.6 (*SD*=13.9); Caucasian 94%; Hispanic 3%.

Bricker et al. (2017) [38]	Before-and-after	USA	Smoked ≥ 5 cigarettes/day for at least 1 year.	General smartphone app.	To employ a single-arm pilot trial of SQ2.0 app to determine participant receptivity, short-term smoking cessation and reduction, and assess the impact of the completion of the SQ2.0 program on smoking cessation and reduction. Furthermore, to explore whether SQ2.0 app is an improvement over SQ1.0 app.	(1) ≥18 years old; (2) smokes at least 5 cigarettes per day for at least 1 year; (3) willing to quit smoking in the next 30 days; (4) daily access to their own smartphone; (5) not using other smoking cessation interventions.	*N*=99 (Female 78%; mean age 38.4; *SD*=8.9); Race: Caucasian 97%; Ethnicity: Hispanic 6%.
Buller et al. (2014) [4~~4~~1]	RCT	USA	Current/daily smoker.	General smartphone app.	To compare, in terms of abstinence and tobacco use, a REQ-Mobile app with text messaging to support smoking	(1) people aged 18–30; (2) current smokers; (3) interested in quitting; (4) US resident; (5) English proficient.	*N*=102 (Female 51%; mean age 24.9); Race: White 74%; African American 14%; Other race 12%; Ethnicity: Hispanic 6%.<br>REQ-MOBILE group (*n*=51): Female 45%; mean age 25.5; Race: White

					cessation in a pilot study.		70%; African American 16%; Other race 14%; Ethnicity: Hispanic 4%. ONQ group (*n*=51): Female 57%; mean age 24.3; Race: White 76%; African American 12%; Other race 12%; Ethnicity: Hispanic 8%.
Businelle et al. (2016) [39]	Before-and-after	USA	Smoking ≥ 5 cigarettes/day.	App combined with face-to-face contact.	To evaluate the feasibility and effectiveness of Smart-T app.	(1) obtained a score of ≥4 on the REALM-SF instrument, indicating higher than sixth-grade English literacy level; (2) willing to quit smoking 7 days after their first clinic visit; (3) ≥18 years old; (4) breath CO of ≥8 parts per million (ppm); (5) smoking at least 5 cigarettes per day; (6) were willing and able to attend 6 weekly assessment sessions and the 12-week follow-up session.	*N*=59 (Female 54%; mean age 52.0; *SD*=7.0); African American 53%.
Carpenter et al. (2015) [40]	Before-and-after	USA	Smoking ≥ 10 cigarettes/day for at least 1 year.	App combined face-to-face contact.	To assess in a pilot study the feasibility of mCM within a smoking cessation intervention	(1) were currently homeless or homeless more than twice in the past year; (2) smokes at least 10 cigarettes per day for at least 1 year and had a CO at the baseline session of at least 10 ppm; (3)	*N*=20 (Female 10%; mean age 54.7; *SD*=7.0).

					among homeless veteran smokers.	were eligible for VA care; (4) people aged 18–70.	
Dan et al. (2016) [5445]	Before-and-after	USA	Reports a minimum 2-year smoking history at a rate of ≥ 10 cigarettes/day.	App combined face to-face-contact.	To evaluate the feasibility, acceptability, and preliminary efficacy of a smartphone-based contingency management program to promote smoking cessation among individuals diagnosed with ADHD.	(1) provides written documentation for their ADHD diagnosis and medical treatment, if applicable, for a continuous period greater than 3 months; (2) ≥18 years old; (3) English-speaking/reading/writing; (4) reports a minimum 2-year smoking history at a rate of 10 or more cigarettes per day; (5) owns a smartphone; (6) breath CO of ≥10 parts per million (ppm); (7) verbally expresses a desire to quit smoking.	*N*=3 (Female 66.66%; mean age 35.66); White 100%.
Dar (2017) [34]	CCT	Israel	Smoking ≥ 5 cigarettes/day.	General smartphone app.	To examine in a pilot study whether monitoring and notifying smokers about smoking episodes in real time via the SmokeBeat app would lead to reduction in smoking.	(1) People aged 18–45; (2) smoked at least 5 cigarettes per day; (3) wished to reduce or quit smoking; (4) owned an android smartphone.	*N*=40 (Female 22.5%); Caucasian 100%. Experimental Group (*n*=20): mean age 25.90 (*SD*=4.22). Control group (*n*=20): mean age 29.55 (*SD*=8.72).

Garrison et al. (2020) [476]	RCT	USA	Smoking ≥ 5 cigarettes/day .	General smartphone app.	To compare the efficacy of Craving to Quit app to reduce smoking rates compared with ES app and to test whether mindfulness training reduced the association between craving and smoking.	(1) People aged 18–65; (2) smoking at least 5 cigarettes per day; (3) had ≤3 months past-year abstinence; (4) own a smartphone; (5) and were motivated to quit, indicated by ≥8/10 on the Contemplation Ladder and ≥4/5 on an Action item of the Readiness to Change Questionnaire: “I am trying to smoke less than I used to,” 1 = strongly disagree, 5 = strongly agree.	*N*=325. MMT-ES Group (*n*=143): Female 72%; main age 43.3 (*SD*=11.1); Caucasian 80%; Hispanic 3%. ES group (*n*=182): Female 71%; main age 39.7 (*SD*=12.6); Caucasian 80%; Hispanic 5%.
Hébert et al. (2020) [412]	RCT	USA	Smoking ≥ 5 cigarettes/day .	App combined face-to-face contact.	To compare in a pilot RCT the feasibility and preliminary effectiveness of Smart-T2 app (experimental group with the QuitGuide app and usual in-person tobacco cessation treatment.	(1) demonstrated an English literacy level greater than the sixth grade; (2) willing to quit smoking 7 days from their first visit; (3) were ≥18 years of age; (4) had an expired carbon monoxide (CO) level >7 ppm; (5) smoking at least 5 cigarettes per day; (6) willing and able to attend four in-person assessment sessions; (7) had no contraindications for nicotine replacement therapy.	*N*=81 (Female 50%; mean age 49.6; *SD*=11.9); White 67%; Black 17%; Other 14%. Usual care group (*n*=27): Female 44%; mean age 51.3 (*SD*=10.1); White 66%; Black 22%; Other 11%. QuitGuide group (*n*=27): Female 59%; mean age 44 (*SD*=12.6); White 70%; Black 22%; Other 7%. Smart-Treatment group

							(*n*=27): Female 48%; mean age 53.6 (*SD*=11.1); White 66%; Black 7%; Other 25%.
Hertzberg et al. (2013) [35]	CCT	USA	Smoking ≥ 10 cigarettes/day for at least 1 year.	App combined face-to-face contact.	To evaluate in a pilot study the feasibility and the efficacy of mCM among smokers with PTSD versus a yoked control mCM.	(1) current PTSD based on the Clinician Administered PTSD Scale; (2) smoked at least 10 cigarettes per day for at least 1 year.	*N*=22. Contingency management group (*n*=11): Female 36.4%; mean age 42.5 (*SD*=14.5). Control group (*n*=11): Female 27.3%; mean age 53.3 (*SD*=11.6).
Hicks et al. (2017) [36]	CCT	USA	Smoking ≥ 10 cigarettes/day.	App combined face-to-face contact.	To evaluate the usability and feasibility of Stay Quit Coach through a pilot study.	(1) Smoked ≥ 10 cigarettes a day; (2) smoked ≥ 1 year; (3) current PTSD based on the Clinician-Administered PTSD Scale; (4) English-speaking/writing; (5) people aged 18-70; (6) willing to quit smoking.	*N*=11. QUIT4EVER Group (*n*=5): Female 80%; mean age 53.2 (*SD*=10.5); Black or African-American 100%. Combine Contact Control Group (*n*=6): Female 50%; mean age 54.3 (*SD*=9.5); Black or African-American 83%; White 17%.
Iacoviello et al. (2017) [43~~9~~]	Before-and-after	USA	Smoking ≥ 5 cigarettes/day.	General smartphone app.	To assess the engagement, efficacy, and	(1) people aged 18-65; (2) smoke at least five cigarettes per day; (3) want to quit smoking in the next 30 days; (4) own a smartphone;	*N*=416 (Female 59.4%; mean age 36; *SD*=10.8); White 75.7%; Hispanic 8.9%; African American

					safety of Clickotine app.	(5) willing and able to receive text messages; (6) able to comprehend the English language; (7) US resident; (8) provide informed consent.	5.3%; Asian or Pacific Islander 2.6%; Native American 1.2%; Other/no response 6.3%.
Janes et al. (2019) [~~50~~49]	RCT	USA	Smoking > 10 cigarettes/day.	App combined face-to-face contact.	To compare outcomes for a Mindfulness training group (experimental group) versus a National Cancer Institute's QuitGuide app group (control group) on PCC reactivity to smoking cues and smoking reduction.	(1) smoking >10 cigarettes/day; (2) <3 months of smoking abstinence in the previous year; (3) 8+ of 10 on a "Readiness-to-change" scale; (4) owns a smartphone; (5) people aged 21-65.	*N*=67 (Female 67.1%; mean age 44.4; *SD*=11). Mindfulness training group (*n*=33): Female 60.6%; mean age 46 (*SD*=11); White 90.90%; Asian 3.03%; Hispanic 3.03%; White Hispanic 3.03%. National Cancer Institute's QuitGuide app group (*n* = 34): Female 73.5%; mean age 43 (*SD*=11); White 91.18%; White Hispanic 5.88%; White & African American 2.94%.
Krishnan et al. (2019) [37]	CCT	USA	Current/daily smokers.	App combined face-to-face contact.	To assess in a pilot study the efficacy of the COach2Quit app in facilitating smoking cessation and the feasibility and acceptability	(1) ≥18 years old; (2) current daily smokers; (3) owns a smartphone; (4) willingness to set a quit date within 2 weeks of the baseline assessment.	*N*=89 (Female 58%; mean age 52); White 17%; Black/African American 79%; Mixed race/other 4%. Intervention group (*n*=39): Female 59%; mean age 53;

					of the COach2Quit app to brief advice.		White 13%; Black/African American 85%; Mixed race/other 2%. Control group (*n*=50): Female 58%; mean age 5; White 20%; Black/African American 74%; Mixed race/other 6%.
Marler et al. (2019) [427]	Before-and-after	USA	Smoking ≥ 5 cigarettes/day.	General smartphone app.	To assess participant engagement, changes in attitudes toward quitting, and changes in smoking behavior in an initial evaluation of the Pivot program.	(1) people aged 18-65; (2) English-speaking; (3) smoke ≥5 CPD; (4) own a smartphone; (5) employed for ≥20 hours a week; (6) US resident.	*N*=319 (Female 57.7%; mean age 42.8; *SD*=10.2); White 82.8%; African American 6.9%; Hispanic 4.7%; Asian 1.6%; American Indian or Alaska Native 1.3%; Native Hawaiian or other Pacific Islander 0.6%; Other 2.2%.
Masaki et al. (2019) [501]	Before-and-after	Japan	Smoke cigarettes with a Brinkman index of at least 200 (only for	App combined face-to-face contact.	To evaluate in a pilot study whether CASC was useful and effective for long-term abstinence in patients with	(1) ≥ 20 years old; (2) diagnosed with a nicotine dependence score of greater than or equal to 5 points on the TDS; (3) smokes cigarettes with a Brinkman index of at least 200 (only for those not younger than 35 years); (4) wishes to quit	*N*=55 (Female 29%; mean age 43.3; *SD*=10.5).

			those not younger than 35 years).		nicotine dependence.	smoking; (5) owns a smartphone; (6) provides informed consent.	
Masaki et al. (2020) [464]	RCT	Japan	Current/daily smokers.	App combined face-to-face contact.	To test whether the addition of the CASC system to a standard smoking cessation program improved long term CARs in patients with nicotine dependence	(1) people diagnosed with a nicotine dependence score ≥ 5 on the TDS; (2) had a smoking history of pack-years ≥10; (3) intended to quit smoking immediately; (4) agreed to participate in a smoking cessation treatment program with written informed consent; (5) could use a smartphone without difficulty.	*N*=572 (Female 25%; mean age 46; *SD*=11) CASC group (*n*=285): Female 24%; mean age 47(*SD*=11). Control group (*n*=287): Female 27%; mean age 45 (*SD*=11).
McClure et al. (2018) [512]	Before-and-after	USA	Be a daily smoker for ≥ 3 months and smoke ≥ 5 cigarettes/day.	App combined face-to-face contact.	To assess the compliance, feasibility, acceptability, and accuracy of M3 app measured during a quit attempt.	(1) people aged 15-25; (2) a daily smoker for ≥ 3 months and smokes ≥ 5 cigarettes per day (CPD) on average; (3) willing to abstain from marijuana or other tobacco products during the study; (4) willing to engage in a brief quit attempt lasting 48 hours.	*N*=16 (Female 75%; mean age 22.3; *SD*=2.0); Race: White 88%; African American/Black 12%; Ethnicity: Hispanic or Latino 6%.
Minami et al. (2018) [523]	Before-and-after	USA	Smoked ≥ 5 cigarettes/day for the past 6 months.	App combined face-to-face contact.	To describe the intervention development and study design and methods for an	(1) ≥18 years old; (2) smoking at least 5 cigarettes per day for at least 6 months; (3) English-speaking; (4) intent to quit smoking in the next 3 months; (5)	*N*=8 (Female 100%, mean age 55.25; *SD*=4.59); Race: White or caucasian 37.5; Black or African American 50%; Asian

					ongoing preliminary randomized controlled trial of Project mSMART MIND. To report the results of an open-label pilot feasibility study and discuss pertinent considerations.	attended a minimum of 3 treatment visits in the past 3 months at the clinic; (6) has a depressive disorder or bipolar disorder; (7) currently receiving treatment for depression or bipolar disorder.	12.5%; Ethnicity: Hispanic 50%.
O'Connor et al. (2020) [45~~3~~]	RCT	Ireland	Smoking ≥ 10 cigarettes/day for at least 1 year.	App combined face-to-face contact.	To evaluate the efficacy of an ACT-based smartphone app in improving ACT group treatment for smoking cessation.	(1) people aged 18 years or older; (2) smoking ≥ 10 cigarettes per day for the past 12 months or more; (3) interested in quitting smoking; (4) willing to engage in 6 weekly group treatment sessions; (5) daily access to a smartphone that was compatible with apps from iTunes or Google Play; (6) not receiving any other treatment to quit smoking.	*N*=150 (Female 52.66%; main age 35.99; *SD*=9.92). Combined group (*n*=50): Female 46%; main age 34.08 (*SD*=10.14). ACT group (*n*=50): Female 50%; main age 35.08 (*SD*=8.72). Behavioral support group (*n*=50): Female 62%; main age 38.80 (*SD*=10.37).
Raiff et al. (2017) [48]	Before-and-after	USA	Smoked ≥ 5 cigarettes/day for at least 1 year.	App combined face-to-face contact.	To evaluate the feasibility of a smartphone-delivered group CM intervention in	(1) English-speaking; (2) aged 19- 48; (3) owns a smartphone; (4) smokes at least 5 cigarettes per day for at least 1 year; (5) intake breath CO of ≥10 parts per million	*N*=10 (Female 50%; mean age 26.2); White 70%; Asian 20%; Not Known 10%.

					smokers who already knew each other.	(ppm); (6) in good physical and mental health; (7) answers affirmatively when asked, “Do you want to quit smoking?”; (8) participants were required to identify one other smoker who wanted to participate and met all of the requirements.	
Wilson et al. (2019) [534]	Before-and-after	USA	Smoking ≥ 10 cigarettes/day for at least 1 year.	App combined face-to-face contact.	To tailor and refine a smoking cessation intervention for the population of smokers diagnosed with schizophrenia, schizoaffective, or psychotic disorder.	(1) smokes at least 10 cigarettes per day; (2) smoking for at least one year; (3) English-speaking/writing; (4) aged 18-70; (5) willing to quit smoking; (6) met criteria for schizophrenia, schizoaffective disorder or another psychotic disorder as determined by the Structured Clinical Interview for DSM-5 Diagnosis.	*N*=13 (Female 38.5%; mean age 47.8; *SD*=11.0); Black or African American 76.92%; White 7.69%; Multiracial 15.38%.

CTC Crush the Crave, *RCT* Randomised Controlled Trial, *CCT* Controlled clinical trial, *MT* Mindfulness training, *PCC* posterior cingulate cortex, *CM* Contingency management, *mCM* Mobile contingency management, *PTSD* posttraumatic stress disorder, *SQ2.0* SmartQuit 2.0, *SSC App* Smartphone Smoking Cessation Application, *ADHD* attention-deficit/hyperactivity disorder, *CASC* CureApp Smoking Cessation, *CO* carbon monoxide, *PPM* parts per million, *REALM-SF* Rapid Estimate of Adult Literacy in Medicine–Short Form, *CPD* cigarettes per day, *TDS* Tobacco Dependence Screener.
