# Supplementary material for: Smoking Cessation Apps: A Systematic Review of Format, Outcomes, and Features
Source: Int J Environ Res Public Health. 2021 Nov 6;18(21):11664. doi: 10.3390/ijerph182111664 (PMC8583115; doi:10.3390/ijerph182111664)
Supplement: Supplementary file 1 [file ijerph-18-11664-s001.zip › Table S3.pdf]

Supplemental Material

Table S3. Intervention descriptions and main outcomes of general smartphone apps.

Author (year)	Intervention description	Control condition description	Tobacco use outcomes	App's features	Conclusion
Baskerville et al. (2018) [55]	CTC group: CTC app.	Control group: OnRQ standard print-based self-help guide with similar content to the CTC app.	ITT continuous abstinence at 6-months point-assessment was not significantly different for CTC group (7.8%) compared to the control group (9.2%).<br><br>However, 7-day PPA at 6 months point-assessment using baseline observation carried forward showed a significant difference in favor of OnRQ (22.3% vs 18.3%).<br><br>Of the completers (n=725) at 6-months point-assessment, 13.8% of the CTC participants and 15.4% of the control group showed continuous abstinence rates.	• CTC app:<br><br>(1) Customize a quit plan: set a quit date and decide whether to quit immediately or reducing.<br>(2) Reminders about the money saved and their health improved.<br>(3) Share their progress with their social network (Facebook and Twitter), and rally support from friends and family.<br>(4) Additional support for quitting: Facebook community.<br>(5) Supportive messages and inspirational photos.<br>(6) Record when, where, and why they were smoking to understand the triggers and psychosocial factors related to smoking.	CTC app was feasible for delivering cessation support but was not superior to a OnRQ self-help guide to quit smoking.

			Furthermore, there was no significant difference in the CPD at 6-month point-assessment between groups.	(7) Graphic and tabular performance feedback.<br>(8) Web-based distractions to help deal with cravings.<br>(9) Evidence-informed information (e.g., relapse and dealing with cravings).<br>(10) Push notifications on rewards received, and helpful reminders to continue to use the app.<br>(11) Access to evidence-based cessation services.	
BinDhim et al. (2018) [32]	Intervention group: smartphone smoking cessation decision-aid app with support features.	Control group: uses an app that contains only smoking cessation information.	Self-reported continuous abstinence at 10-day point-assessment (32.2% vs. 20.8%), 1-month point-assessment (28.5% vs. 16.9%), 3-month point-assessment (23.8% vs. 10.2), and 6-month point-assessment (10.2% vs 4.8%) significantly increased in the intervention group compared to the control group.	• The intervention app:<br>(1) Set a quit date.<br>(2) Mandatory information about quitting options.<br>(3) Daily motivational messages.<br>(4) A quitting diary.<br>(5) A quitting benefits tracker.<br>• The control app:<br>(1) Smoking cessation information.<br>(2) Set a quit date.	The smartphone decision-aid app significantly increased smoking cessation rates.

				(3) Included non-mandatory information about quitting options, benefits, and harm.	
Bricker et al. (2014) [33]	SmartQuit group: SmartQuit app.	QuitGuide group: The NCI ́s “QuitGuide” app.	SmartQuit group had higher quit rates at 2-month post-enrollment point-assessment compared to QuitGuide group (13% vs. 8%).	• SmartQuit:<br>(1) Motivations to quit via testimonials and photos uploaded by users.<br>(2) A personalized quit plan, identifies social support and provides information about medications for quitting.<br>(3) ACT audio and text-based acceptance skills for coping with cravings to smoke.<br>(4) ACT skills in self-compassion for recovering from smoking lapses and self-judgments.<br>(5) Track urges included self-monitoring cigarettes smoked and medication.<br>Main menu: view and update their quit plan, view their progress, receive graphic rewards, or share their progress.<br>• The NCI´s “QuitGuide” app:	ACT is feasible to deliver by smartphone app, acceptable for the majority of users, and shows promising quit rates compared to an app that follows US Clinical Practice Guidelines.

				(1) Reason-based motivations to quit.<br>(2) A personalized quit plan, identifies social support and provides information about medications for quitting.<br>(3) Skills for avoiding cravings to smoke.<br>(4) Skills for coping with lapses via fighting cravings and trying to be positive.	
Bricker et al. (2017) [38]	SQ2.0 app.	No comparison group.	The quit rates at 2-month-post-enrollment point-assessment were 21% for 7-day PPA, 11% for 30-day PPA, and the smoking reduction rate was 75%.<br><br>Smoking cessation (33% for 7-day PPA, 28% for 30-day PPA) and reduction rates (88% of participants) were higher among those who completed the program.	• SQ2.0 app:<br><br>(1) Quit plan.<br>(2) Eight core ACT exercises.<br>(3) Track urges.<br>(4) Other ACT-based exercises to support quitting, and additional exercises to reinforce content presented in the core exercises.<br>(5) Certificate of completing the program.	SQ2.0 had high user receptivity, modest quit rates, and high reduction of smoking.

Buller et al. (2014) [441]	REQ-Mobile group: REQ-Mobile app.	onQ group: onQ text messaging system (tasks to plan, set, and maintain a quit date, cope with cravings and relapse, and consolidate a nonsmoking lifestyle).	Significantly more onQ smokers had quit at 6-week-posttest point-assessment than REQ-Mobile smokers.<br><br>Of the completers at 6-week-posttest point-assessment (n=66), 58% of the ONQ group participants and 30% of the REQ-Mobile group participants had 7-day PPA.<br><br>Of the completers at 12-week-posttest point-assessment (n=68), 46% of the ONQ group participants and 27% of the REQ-Mobile group participants had a 30-day PPA. Of the ITTsample at 12-weeks posttest point-assessment (n=102), 31% of the ONQ group participants and 18% of the REQ-Mobile group participants had a 30-day PPA.	• REQ-Mobile:<br><br>(1) Short messages.<br>(2) Audio phase-transition messages to enhance comprehension.<br>(3) Participants could create lists of reasons for and benefits of quitting and plans for coping with challenging situations and stressful circumstances.<br>(4) Listen to 13 audio testimonials from former smokers.<br>(5) Read 16 short support documents.<br>(6) Set a quit date and update the expert system.<br>(7) Short messages directed smokers to quitting tools.<br>(8) 56 messages encouraged them to use the study Website to view additional resources on smoking cessation.	Abstinence rates were lower in the REQ-Mobile group compared to ONQ group. Smokers provided highly favorable evaluations of REQ-Mobile's usability.

			At 12-week-posttest point-assessment, 40% of the completers in the ONQ group and 24% of the RE Q-Mobile group had continuous abstinence. Of the Intent-to-treat sample at 12-week posttest point-assessment, 27% of the ONQ group and 16% of the REQ-Mobile group had continuous abstinence.		
Dar (2017) [34]	Experimental group: (1) smartwatch and (2) SmokeBeat app.	Control group: Wait-list control condition. They did not receive a smartwatch.	There was a significant reduction of CPD in 30-day period (end of the study) in the experimental group compared to the control group.	• SmokeBeat app:<br>(1) Detection of a smoking episode through a real-time detection of hand-to-mouth gestures with a smartwatch.<br>(2) Pop-up notifications of smoking episodes and cigarette follow-up questions.<br>(3) When the episode was not detected, the participant should report having smoked.<br>(4) The participants could search for statistical information regarding their smoking patterns.	The study provides preliminary evidence that automatic monitoring of smoking episodes and notifying the smoker in real time when smoking episodes are detected can lead to smoking reduction.

Garrison et al. (2020) [476]	MMT-ES group: Craving to Quit app.	ES group: ES app.	At 6-month point-assessment, one-week PPA biochemically verified was 9.8% in the MMT-ES group and 12.1% in the ES group- Furthermore, this one-week PPA and continuous abstinence did not differ between groups.<br><br>Additionally, from baseline to 6-month point-assessment, there was an overall reduction in CPD. This reduction did not differ between conditions.	• Craving to Quit<br><br>(1) 22 days of training modules (5–15 minutes/day) teaching mindfulness.<br>(2) Teaching mindfulness and three standard meditation practices: body scan, loving-kindness, and breath awareness.<br>(3) Teaching an informal practice to work mindfully with cravings, RAIN: Recognize, Accept, Investigate, and Note what cravings feel like as they arise and pass away.<br>(4) ES feature: “check-ins” to measure smoking, craving, and other factors. Users were asked to check-in six times per day for 22 days.<br><br>• App control group<br><br>(1) ES feature: “check-ins” to measure smoking, craving, and other factors for 22 days.	Smartphone app-based mindfulness training may help to decrease the association between craving and smoking.

Iacoviello et al. (2017) [439]	Clickotine app.	No comparison group.	Of the study sample (ITT), at the end of the study (8-week study period), 45.2% of participants reported 7-day abstinence and 26.2% of participants 30-day abstinence.<br><br>Of the study completers (n=365) 51.5% reported 7-day abstinence and 29.9% 30-day abstinence.	• Clickotine app.<br><br>(1) A quit date.<br>(2) Delivery of intervention components: controlled breathing, personalized messaging, social engagement, encouragement of pharmacotherapy for cessation and of medication adherence, and digital diversions.<br>(3) Interactions for the user: controlling breathing, using digital diversions, logging cravings, receiving personalized messages, responding to messages, participating with “quit teams” completing missions, logging emotions and feelings, logging cigarettes smoked, journaling, learning about and using quit aids, and interacting with supporters.	Participants engaged with the app and appeared to remain engaged with the app for most of the study duration on average. Clickotine use may be associated with cessation outcomes.
Marler et al. (2019) [427]	(1) CO breath sensor, which communicates with a smartphone and app, (2) the Pivot mobile app and (3)	No comparison group.	Of the study sample (ITT), at the end of the study, 32.0% achieved 7-day PPA, and 27.6% achieved 30-day PPA.	• Pivot mobile app:<br><br>(1) Log cigarettes.<br>(2) Complete in-app activities.	Pivot was shown to be engaging, and quit rates were aligned with those in the peer-reviewed literature. This was true

dedicated human coaching delivered one-on-one through in-app text messaging.	Of the study completers, 37.5% (n=272) achieved 7-day PPA, and 32.4% achieved 30-day PPA. Among the study completers who did not achieve at least 7-day PPA, CPD were reduced by 29.1%.	(3) Interact with their coach via in-app chat.<br>(4) Breath samples.	both in individuals who were and were not ready to quit in the next 30 days, with comparable retention and cessation outcomes between the 2 groups (ready to quit and not ready to quit).

CTC Crush the Crave, *OnRQ* On the Road to Quitting, *ITT* intention to treat, *PPA* point prevalence abstinence, *CPD* cigarettes per day, *NCI* National Cancer Institute, *US* United States, *SQ2.0* SmartQuit 2.0, *ACT* Acceptance & Commitment Therapy, *REQ-Mobile* The Real e Quit mobile application, *MMT-ES* mobile mindfulness training with experience sampling, *ES* experience sampling only, *CO* carbon monoxide.
