# Supplementary material for: Smoking Cessation Apps: A Systematic Review of Format, Outcomes, and Features
Source: Int J Environ Res Public Health. 2021 Nov 6;18(21):11664. doi: 10.3390/ijerph182111664 (PMC8583115; doi:10.3390/ijerph182111664)
Supplement: Supplementary file 1 [file ijerph-18-11664-s001.zip › Table S4.pdf]

Supplemental Material

Table S4. Intervention descriptions and main outcomes of apps combined with face-to-face contact.

Author (year)	Intervention description	Control condition description	Tobacco use outcomes	App's features	Conclusion
Businelle et al. (2016) [39]	(1) usual care and (2) Smart-T app.	No comparison group.	A total of 41% of participants were abstinent at quit date,17% met criteria of 7-day PPA at 1-week point-assessment, 31% at 2-week point-assessment, 27% at 3-week point-assessment, 22% at 4-week point-assessment, and 20% at 12-week point-assessment. The proportion of abstinent participants was significantly higher at the 2-week point-assessment compared with the 1-week point-assessment.	• The Smart-T app:<br>(1) EMAs.<br>(2) Automated Messages.<br>(3) On-Demand Content: direct link to Quitline, smoking cessation messages and medication information.<br>(4) Direct link to study staff.<br>(5) EMAs information.<br>(6) Daily message.	This app, used with socioeconomically disadvantaged smokers who received a smoking cessation treatment, was useful and helpful. Furthermore, the rate of abstinence was higher than other treatments for this population.
Carpenter et al. (2015) [40]	(1) 7 face-to-face visits (1 assessment session, 4 counseling sessions and 2 follow-up sessions), (2) 1 brief telephone session, (3) 5 weeks	No comparison group.	The quit rate was 50% abstinence rates at 4-week point-assessment. Abstinence was maintained in 65% at 3-month point-assessment and 60% at 6-month point-assessment.	• mCM app:<br>(1) Generate a side-profile video recording of themselves recording their CO.<br>(2) Log into a secure website.	mCM is feasible and may contribute to increases in initial and long-term quit rates as part of a multicomponent smoking cessation intervention for homeless smokers.

	of CO monitoring with mobile CM app (CM for negative CO samples <6 ppm), and (4) pharmacotherapy.			(3) Upload video recordings confirming abstinence.<br>(4) Receive compensation information.	
Dan et al. (2016) [5445]	(1) Daily videos measuring participants CO (CO Smokerlyzer + smartphone app Motiv[8] Systems) and (2) Monetary vouchers for reinforcement smoking abstinence (CM) through negative CO samples (≤4 ppm).	No comparison group.	There was an increase in the percentage of abstinence during different point-assessments. All participants had smoked at 1-week follow-up.	• Motiv[8] app:<br>(1) Self-recorded CO levels.<br>(2) View earnings.<br>(3) View a graph of their progress.	This app for smokers with ADHD had good acceptability and feasibility results.
Hébert et al. (2020) [412]	Smart-Treatment: (1) Smart-T2 app, (2) four laboratory visits and (3) 2-week supply of over-the-counter NRT for the initial post-quit period.	NCI group: (1) NCI QuitGuide app, (2) four laboratory visits and (3) 2-week supply of over-the-counter NRT for the initial post-quit period.	At 4 weeks point-assessment (post-quit date), a total of 26% of participants were confirmed abstinent (Smart-T2: 22%, QuitGuide: 26%, usual care: 30%), and, at 12-weeks point-assessment (post-quit date), 17% of participants were confirmed abstinent (Smart-T2: 6/27,	• Smart-T2 app:<br>(1) Tailored messages to help participants cope.<br>(2) “Quit Tips” button offering cessation advice, coping strategies, and quitting benefits.<br>(3) “Medications” button offering information about	Smoking cessation treatments that use smartphone apps may be capable of providing similar outcomes to traditional treatments like face-to-face counseling.

		Usual care group: (1) six weekly individual counseling sessions from 1 week before the quit date through 4-week-post-quit date, (2) 2-week supply of over-the-counter NRT for the initial post-quit period.	22%, QuitGuide: 4/27, 15%, usual care: 4/27, 15%).<br><br>There were no significant differences in smoking abstinence between conditions.	smoking cessation medications.<br>(4) "Phone a Counselor" button that calls the free Oklahoma Tobacco Help Line.<br>(5) Daily treatment messages (e.g., your quit date is tomorrow).<br>(6) A button to request additional NRT through the EMA app home screen.<br><br>• The NCI QuitGuide app:<br><br>(1) Strategies for quitting.<br>(2) Health outcome information.	
Hertzberg et al. (2013) [35]	mCM group: (1) two face-to-face smoking cessation counseling sessions (including controlled breathing training, short relaxation strategies, examination of smoking triggers, and preparation for the quit day) based on the NCI ́s Freshstart program, (2) NRT, (3)	Yoked control group: (1) two smoking cessation counseling sessions (including controlled breathing training, short relaxation strategies, examination of	82% (n=9 of 11) in the mCM group and 45% (n=5 of 11) in the yoked group had quit smoking at 4-week point-assessment.<br><br>At a 3-month point-assessment, abstinence rates were 55% in the mCM group.	• mCM app:<br><br>(1) Generate a side-profile video recording of themselves recording their CO.<br>(2) Log into a secure website.<br>(3) Upload video recordings confirming abstinence.<br>(4) Receive compensation information.	Feasibility of mCM for smoking cessation among smokers with PTSD was higher than yoked control group.

	use of low-nicotine cigarettes 2 weeks before their quit day, (4) bupropion, and (5) CO monitoring and CM for CO reductions in tapering condition and for negative CO samples (≤8 ppm) through an mCM app.	smoking triggers, and preparation for the quit day) based on the NCI ́s Freshstart program, (2) NRT, (3) low-nicotine cigarettes, (4) Bupropion, and (5) CO monitoring and Noncontingent management through an mCM app.			
Hicks et al. (2017) [36]	QUIT4EVER group: (1) mCM app: CO measures and CM for negative CO samples (≤6 ppm), (2) smoking cessation counseling (workbook and four 20-minute CBT sessions), (3) medications (NRT and bupropion), and (4) Stay Quit Coach app.	Combine Contact Control (CCC) group: QUIT4EVER except for Stay Quit Coach app.	Abstinence rates at post-treatment were 60% in the QUIT4EVER and 100% in the Combined Contact Control. At 2-week point-assessment, post-treatment abstinence rates were 60% in the QUIT4EVER and 67% in the Combined Contact Control.	• mCM app:<br>(1) Generate a side-profile video recording of themselves record their CO.<br>(2) Log into a secure website.<br>(3) Upload video recordings confirming abstinence.<br>(4) Receive compensation information.<br>• Stay Quit Coach app:	Combined use of mCM and Stay Quit Coach is a feasible and acceptable adjunctive smoking cessation treatment among smokers with PTSD.

			Three participants of the QUIT4EVER group reported prolonged abstinence at 3- and 6-month point-assessments, and another three participants of the CCC group reported prolonged abstinence only at 3-month point-assessment.	(1) Brainstorming reasons for quitting.<br>(2) Interactive tools to help cope with smoking urges and lapses.<br>(3) Motivational messages.<br>(4) Support contacts.	
Janes et al. (2019) [~~50~~49]	MT group: 1-month intervention (app-based MT program).	NCI group:1-month intervention (NCI QuitGuide app).	There was a reduction in CPD in both conditions from baseline to 1-month follow-up. The reduction in cigarettes/day was greater for MT group (average reduction=11.4 ± 7.4) than for NCI group (average reduction=8.7 ± 5.8).	• App-based MT program:<br>(1) Cover habit formation by positive and negative reinforcement.<br>(2) Reinforce prior modules using animations and cover recognition of triggers and how to use noting practice in everyday life.<br>(3) Build on RAIN and noting practice by teaching curiosity, a core element of mindfulness.<br>(4) Covers loving-kindness meditation.<br>(5) Covers the costs and benefits of smoking.<br>(6) Work mindfully with thoughts that trigger smoking.	Using app-delivered MT, a reduction in cue-induced posterior cingulate cortex reactivity predicted a concomitant reduction in cigarette smoking, an effect that was specific to individuals in the MT group.

				(7) Ask participants to reflect on their own experience with the app.<br>(8) Quit Day ceremony.<br>(9) Mindfulness practices.<br><br>Other features:(a) daily check-ins, (b) cigarette tracker, (c) night reflection, and (d) a quitting pact.<br><br>• The NCI QuitGuide app:<br><br>(1) Strategies for quitting.<br>(2) Health outcomes information.	
Krishnan et al. (2019) [37]	Intervention group: (1) brief advice along with the (2) CO monitor and (3) COach2Quit app.	Control group: brief advice.	At 30-day point-assessment, one participant in each study arm (3% vs. 2%) had quit smoking. There were no significant differences between study arms in median changes in CO levels or CPD between baseline and 30-day point-assessment.	• COach2Quit app:<br><br>(1) Set a quit date.<br>(2) Remind the user to take a CO breath test.<br>(3) Response messages to the user based on CO result.<br>(4) A graphical display of their CO readings.<br>(5) Instructions to stop smoking.<br>(6) Contact information for help.	No significant differences in smoking cessation, smoking reduction, and motivation to quit smoking between study arms over a 30-day period.
Masaki et al. (2019) [~~51~~50]	(1) CASC smartphone app, (2) advice to the participants during	No comparison group.	The continuous abstinence rate from weeks 9 to 24 point-assessments was 64%,	• CASC smartphone app:	The addition of CASC to usual smoking cessation therapies resulted in high

	their clinic visits and CO measures.		from weeks 9 to 12 point-assessments and 9 to 52 weeks point-assessments were 76% and 58%, respectively.	(1) Diary of smoking cessation: cessation status, physical condition, medication use, and adverse events.<br>(2) Messages and educational videos to help users quit smoking.<br>(3) Counseling chat sessions between the users and the artificial intelligence nurse.	continuous abstinence rates, high patient retention rates, and improvement of cessation-related symptoms.
Masaki et al. (2020) [4446]	CASC group: (1) 12 weeks of standard smoking cessation treatment (pharmacotherapy and counseling), (2) CASC system for 24 weeks (smartphone app, connected cloud system to upload data, paired mobile exhaled CO checker device, and web-based PC software for physicians).	Control group: (1) 12 weeks of standard smoking cessation treatment with pharmacotherapy and counseling, (2) control-app (basic functions of the CASC app) without a mobile CO checker for 24 weeks.	The continuous abstinence rate from weeks 9 to 12 weeks point-assessments (75.4% vs 66.2%), 9 to 24 point-assessments (63.9% vs 50.5%) and 9 to 52 weeks point-assessments (52.3% vs 41.5%) were significantly higher in the CASC group than in the control group.<br><br>Time to the first lapse after the quit date was significant higher in the CASC group compared to the control group (median, 267 days vs 19 days)	• CASC smartphone app:<br>(1) Diary of smoking cessation: cessation status, physical condition, medication use, and adverse events.<br>(2) Educational video tutorials to help users quit smoking.<br>(3) Counseling chat sessions<br>(4) Collection and recording breath CO.<br><br>• Control-app:<br>(1) User guide: how to use the app.<br>(2) Participants' profiles.<br>(3) Set a quit date.	The CASC system combined with a standard smoking cessation treatment, significantly improved a long-term continuous abstinence rate from weeks 9 to 24 in patients with nicotine dependence.

				(4) Schedule of five visits with a summary of objectives of each visit.<br>(5) Date of the next appointment.<br>(6) Contact form for technical support.<br>(7) App version, privacy policy, and administrative information.	
McClure et al. (2018) [~~52~~51]	(1) face-to-face visits (motivational enhancement, brief cessation counseling and cessation materials), (2) M3 app for monitoring CO and EMA with CM for negative CO samples (≤6 ppm).	No comparison group.	25% of the participants were abstinent during the entire 2-quit day attempt.	• M3 app:<br>(1) Log cigarette.<br>(2) EMAs.<br>(3) Collection of breath CO.	The use of remote monitoring could help to improve some limitations related to traditional face-to-face treatment.
Minami et al. (2018) [~~53~~52]	(1) individual counseling sessions (Psychoeducation, smoking cessation counseling, and an introduction to the theory and practice of mindfulness, including instructions	No comparison group.	At 2-week, 4-week, and 3-month point-assessment (post-quit date), 1 participant (12.5%) reported biochemically verified 7-day PPA. All participants reported reductions in CPD from baseline to 2-week, 4-	• The app features:<br>(1) EMA reports.<br>(2) Engage in mindfulness practice.<br>(3) Complete a post-mindfulness practice report.<br>(4) Videotape themselves testing their CO levels twice a day.	The results from the feasibility study indicated high levels of acceptability and satisfaction with SMI-CM intervention.

	and feedback), (2) a smartphone intervention app (daily mindfulness practice, smoking status verification for CM, and daily reports), and (3) 8-week supply of nicotine patches.		week, and 3-month point-assessments (post-quit date).	(5) Track incentives accumulated.	
O’Connor et al. (2020) [~~43~~45]	Combined group: (1) SmartQuit app (2) ACT face-to-face treatment (6 weekly 90 mins sessions).	ACT group: ACT face-to-face treatment (6 weekly 90-min sessions).<br><br>Behavioral support group: 6 weekly 90-min sessions according to an evidence-based treatment protocol.	At post-treatment, the biochemically verified quit-rate was 36% in the combined treatment, 20% in the ACT group, and 24% in the behavioral support group.<br><br>At 6-month follow-up, the biochemically verified quit-rate was 24% in the combined group, 24% in the ACT group, and 20% in the behavioral support group.<br><br>At post-treatment and 6-month follow-up, non-abstinent participants in the combined group reported a	• SmartQuit app<br><br>(1) A personalized quit plan.<br>(2) Record why quitting smoking is deeply important (e.g., setting a positive example for the children)<br>(3) Upload a photo to symbolize this value (e.g., picture of the children).<br>(4) 8 core ACT exercises.<br>(5) Track each urge that passed without smoking.<br>(6) 43 exercises, stories, and tips in the app’s anytime coaching section.	Combined a face-to-face treatment with an app (combined treatment) could be efficacious to promote smoking reduction at post-treatment.

			significant reduction in CPD.<br><br>At post-treatment, non-abstinent participants in the combined group reported significantly fewer CPD than the other treatment conditions.		
Raiff et al. (2017) [48]	(1) The NCI´s “Clearing the Air” booklet, (2) Instructional manuals, (3) group social support forum (Kakao talk app), (4) CO measures (mōtiv^{8} app + hand-held breath CO meter).<br>and monetary contingency reinforcement for negative CO samples (≤4 ppm).	No comparison group.	7 participants (n=10) reducing their mean breath CO levels by about 50% from baseline during the intervention. The percentage of negative samples (CO ≤ 4 ppm) increased from a mean of 1% during baseline to 36% during treatment. At 1-month follow-up, all participants had smoked.	• Kakao Talk:<br><br>(1) Group social support forum.<br><br>• Mōtiv^{8}:<br><br>(1) Self-recorded CO levels.<br>(2) View earnings.<br>(3) View a graph of their progress.	CM intervention reduced smoking use.
Wilson et al. (2019) [~~54~~53]	(1) mCM Smartphone App: CO measures and CM for negative CO samples (≤6	No comparison group.	Cohort 1: At post-treatment, 2 participants (40%) reported abstinence. At 3-month follow-up, one	• mCM smartphone app:	Both participants and therapists reported that the intervention was helpful in people with psychotic

	ppm), (2) Stay Quit Coach Smartphone App, (3) Pharmacotherapy and (4) Cognitive Behavioral Counseling (manual and workbook).		participant (20%) reported abstinence. Moreover, one participant reduced CPD at post-treatment.<br><br>Cohort 2: At post-treatment, three participants (38%) reported abstinence. At 3-month follow-up, one participant (15%) reported abstinence. Moreover, three participants reduced CPD at post-treatment.	(1) Generate a side-profile video recording of themselves recording their CO.<br>(2) Log into a secure website.<br>(3) Upload video recordings confirming abstinence.<br>(4) Receive compensation information.<br><br>• Stay Quit Coach Smartphone app:<br><br>(1) Brainstorming reasons for quitting.<br>(2) Interactive tools to help cope with smoking urges and lapses.<br>(3) Motivational messages.<br>(4) Support contacts.	disorders. Over one-third of participants self-reported abstinence at post-treatment.

MT Mindfulness training, *fMRI* functional magnetic resonance imaging, *NCI* National Cancer Institute, *RAIN* Recognize, Accept, Investigate, Note, *CM* Contingency management, *CO* carbon monoxide, *mCM* Mobile contingency management, *NRT* nicotine replacement therapy, *PTSD* Smokers with posttraumatic stress disorder, *CBT* cognitive behavioral therapy, *PPA* point prevalence abstinence, *SMI-CM* smartphone-assisted mindfulness smoking cessation intervention with contingency management, *EMA* ecological momentary assessment, *ADHD* Attention-Deficit/Hyperactivity Disorder, *CASC* CureApp Smoking Cessation, *CPD* cigarettes per day.
